# End-Effector Robotic Rehabilitation After Spinal Cord Injury: A Systematic Review of Devices, Operational Characteristics Used, Outcome Measures, and Rehabilitation Effectiveness

**DOI:** 10.3390/bioengineering13101138

**Published:** 2026-09-29

**Authors:** Michael Baldock, Silvia Caggiari, Antonio Capozio, David E. Lunn, Frances Gawne, Ioannis Delis, Sarah L. Astill

**Affiliations:** 1 School of Biomedical Sciences, Faculty of Biological Sciences, University of Leeds, Leeds LS2 9JT, UK; 2 Department of Mechanical Engineering, School of Engineering, University of Southampton, Southampton SO17 1BJ, UK; 3 Department of Psychology, Faculty of Arts and Sciences, Edge Hill University, Ormskirk L39 4QP, UK; 4 Carnegie School of Sport, Leeds Beckett University, Leeds LS6 3QS, UK

**Keywords:** spinal cord injury, end-effector, kinematics, lower-limb, upper-limb, rehabilitation

## Abstract

Background: End-effector robotic devices are increasingly used in rehabilitation to retrain neural pathways through repetitive, task-specific exercise. However, the influence of movement kinematic variables remains unclear. This systematic review aimed to; (i) identify end-effector robotic systems used in upper- and lower-limb spinal cord injury rehabilitation, (ii) describe their technical specifications and operational use, and (iii) explore associations between device use characteristics and study outcomes. Methods: A systematic review of five databases was conducted from inception to June 2026. Results: Fifteen studies, ten lower-limb and five upper-limb, met the inclusion criteria. These covered five commercially available lower-limb devices and three upper-limb devices. Most lower-limb studies demonstrated moderate-to-large improvements in balance, ambulation (WISCI-II), walking endurance (6mWT), and independence (SCIM-III). End-effector training produced comparable outcomes to both conventional therapy and exoskeleton-based rehabilitation in controlled comparisons. Upper-limb studies showed substantial heterogeneity in outcome selection, reflecting diverse rehabilitation goals. Conclusions: WISCI-II and 10MWT were the most used lower-limb functional assessments, with SCIM-III used to assess independence for both upper- and lower-limb motor impairments. Reporting of kinematic settings are often limited and concentrated at study onset, limiting assessment of their contribution to outcomes. Reporting the progression of session-level metrics is needed to better understand how time-varying kinematics affect intervention effectiveness.

## 1. Introduction

Spinal cord injury (SCI) is a neurological disorder arising from direct or indirect damage to the spinal cord. In the UK alone, between 2500 and 4400 new cases are reported every year, and approximately 50,000 to 105,000 people are currently living with SCI [1]. The vast majority of new injuries are classified as traumatic, and their reported incidence varies between 12.1 and 57.8 cases per million inhabitants in high-income countries [2]. SCI places a substantial financial burden on healthcare systems, with a lifetime cost estimated to be approximately £1.12 million per annum per case [3], depending on the severity of the injury. Individuals with SCI present with a range of motor and sensory deficits, which compromise their physical, psychological, and social wellbeing, resulting in reduced independence and a reduction in their quality of life [4]. Injuries progress from an acute stage (<1 year) to a chronic stage (>1 year) and can be categorised as incomplete or complete depending on the level of damage [5]. The severity of injury is classified by the American Spinal Injuries Association Impairment Scale (ASIA) [6], which includes ASIA A, in which the motor and sensory functions are absent due to a complete injury; B, in which sensory functions are preserved below the neurological level of injury but motor functions are not present; C, in which sensory and motor functions are preserved but over half the key muscles are unable to move against gravity; D, in which sensory and motor functions are preserved but over half the key muscles are able to move against gravity; and E, in which sensory and motor functions are normal.

Spinal cord injury commonly leads to impaired motor function, reduced ambulation, and upper-limb function, depending on the level and extent of the lesion. Higher-level injuries are associated with more widespread functional deficits: cervical injuries (C1–C8) typically impair motor control and sensation in both upper and lower extremities, whereas thoracic (T1–T12) and lumbar (L1–L5) injuries predominantly affect trunk stability and lower-limb function.

Over recent decades, substantial efforts have focused on recovery-orientated approaches aimed at preserving and restoring both lower and upper extremity function following a SCI. Repetitive and activity-based interventions are well established to support motor (re)learning and promote neurological recovery through activity- dependent plasticity within the brain and spinal cord [7]. Robotic-assisted rehabilitation facilitates high intensity, task-specific, and repetitive training and has been shown to enhance the effectiveness of conventional physical therapy [8], which is often limited by insufficient training intensity as well as variability and clinician burden.

With advances in robotics and computing, robotic devices are being developed and implemented in rehabilitation to enhance mobility, function, and ultimately quality of life in individuals with SCI [8,9]. These devices typically include exoskeletons and end-effectors. Exoskeletons require a direct correspondence between robotic and patient anatomical joints, guiding movement along pre-programmed trajectories through the application of mechanical power at specific joints. These devices move with the patient’s skeleton, limiting the freedom of movement outside the programmed trajectories [10]. By contrast, end-effectors generate movement through programmable actuation of the most distal segment (e.g., footplates or handles) without requiring alignment between the robot and anatomical joints. Such systems can deliver pre-defined trajectories that aim to replicate natural limb movements, such as gait cycles in lower-limb applications [11], while allowing free movement of more proximal joints. This unconstrained joint control can contribute to postural control and sensory integration processes through destabilisation training [12,13].

A substantial body of literature has investigated robotic-assisted rehabilitation in individuals with SCI, with the majority focussing on recovering walking function and activity primarily using an exoskeleton device [14]. Studies have been conducted to compare end-effectors and exoskeletons for both upper- and lower-extremity rehabilitation [15,16], and differences in the design, nature of working, cost, and challenges in rehabilitation were highlighted. End-effectors appear to have a simpler design structure and less complicated control algorithms that drive motion [17]. Additionally, the bodyweight support (BWS) offered by end-effector devices may reduce muscle fatigue, while their design and operational principles allow greater adaptability to individual anthropomorphic characteristics and rehabilitation needs. However, considerable variation exists across end-effector models, with increasing degrees of freedom at the footplate and the capability to support both gait and balance training [17]. These enhanced functionalities are typically associated with greater device complexity and cost. However, standardised protocols for their implementation in rehabilitation are currently lacking.

Upper- and lower-limb rehabilitation differ in practice due to the distinct functional demands at each extremity. Upper-limb tasks comprise more dexterous and sensory based activities and are often unilateral, whereas lower-limb tasks are generally bilateral and cyclical in nature. These functional differences can be seen in the stronger and lateralised cortical connectivity for upper-limb movements [18], and increased dependence on the central pattern generator for primitive lower-limb tasks [19]. Furthermore, these differences influence the design of end-effector devices, with upper-limb requiring fine motor control and object manipulation, and lower-limb targeting gait retraining and balance.

In lower-limb research, reproduction of kinematic movement patterns using end-effector gait trainers in healthy individuals has been shown to elicit similar rhythmic muscle activation patterns, albeit with delayed onset and reduced amplitude, particularly in distal musculature near the site of end-effector contact [20,21]. Thus, these findings support the use of kinematic replication as a promising strategy for retraining neural pathways through repeated activation and engagement of existing motoneuronal networks.

However, the speed of task execution influences both movement kinematics and the magnitude of muscle activation [22,23]. For example, walking with a step length or cadence that differs from an individual’s nominal values changes lower-limb kinematics, with the vastus medialis having been shown to be more affected by step length changes, whereas the soleus is more affected by changes to cadence [23]. Furthermore, including BWS can also reduce muscle activation in some muscles but not others [24]. Combined, the implications of how these factors influence the engagement of existing motoneuronal networks, and for rehabilitation in individuals with spinal cord injury, remains unclear.

Therefore, translation to clinical rehabilitation is challenging and further complicated by pre-injury movement patterns being unknown, and secondary impairments such as spasticity and muscle atrophy after injury may restrict joint range of motion and movement velocity. This review aims to provide an overview on the operational characteristics of end-effector devices in lower- and upper-limb rehabilitation in SCI. In particular, the objectives are to:

- Identify end-effector robotic systems used as adjunct rehabilitation tools for individuals with SCI, covering both upper- and lower-limb applications.
- Describe the technical specifications and operational characteristics used for end-effector devices throughout the therapeutic studies.
- Evaluate effectiveness of end-effector devices through analysing functional outcome measures.

## 2. Materials and Methods

This review was conducted in accordance with the Preferred Reporting Items for Systematic Reviews and Meta-Analyses (PRISMA) statement [25], and it was registered with the International Prospective Register of Systematic Reviews (PROSPERO, registration number CRD42024534938).

### 2.1. Data Sources and Searches

A literature search was conducted from inception to April 2025 on Web of Science, MEDLINE, EMBASE, PubMed, and CINAHL, and the search terms (reported in Supplementary Material Section S1) were carefully selected to be appropriate for each of the databases. The search was updated June 2026 for publication. The study selection process was then managed using a recommended web-based systematic review tool [26], Rayyan, which facilitated duplicate removal, study selection and collaboration between researchers. The updated search yielded records matching those from the original search due to the database restriction to year-only searching.

### 2.2. Inclusion and Exclusion Criteria

For this review, an end-effector was defined as a device through which movements are generated from the most distal segment of the extremity, with no alignment between patient-device joints (i.e., footplates or handles used to generate motion for lower or upper limbs in space) [16]. Given the limited available literature, this review includes interventions which involve at least one session with an end-effector for either lower- or upper-limb rehabilitation in individuals with acute or chronic SCI, any level of injury and traumatic or non-traumatic aetiology. Randomised control trials (RCTs) and non-randomised control trials comparing interventions including end-effector use to a comparator intervention, cohort studies, case series and case reports evaluating pre-and post-intervention with end-effector use, or the same intervention in two different populations were selected. Studies using concurrent interventions were also included if at least one employed an end-effector, for example, an intervention with an end-effector in combination with functional electrical stimulation (FES).

In addition, the considered reported outcomes were associated to clinical and biomechanical parameters for the assessment of rehabilitative interventions. Systematic reviews, conference proceedings and articles not published in English were excluded. Studies were also excluded if they involved animals, individuals under 16 years or above 70 years of age, or robotic devices requiring alignment between robotic and anatomical joints.

Two authors (SC and AC) independently performed title and abstract screening for inclusion (April 2025). A manual search was also performed screening the references of the studies selected for inclusion, and the website of each end-effector manufacturer was also checked. Disagreements during this process were resolved through discussion until the authors reached a consensus. The updated search (June 2026) was similarly completed by two authors (AC and MB).

### 2.3. Quality Assessment

SC and AC independently conducted quality assessments for the articles selected for inclusion using the modified version of the Cochrane Risk of Bias Tool previously designed by the authors [27] to accommodate studies with different designs, including RCTs and case series. Discrepancies in scoring were resolved by reassessing each paper and reaching a consensus.

Briefly, the risk of bias tool consists of six domains, each comprising one or more items: (1) selection bias arising from study design, random sequence generation, allocation concealment; (2) performance bias arising from blinding of participants and personnel, concurrent intervention or unintended exposure; (3) detection bias arising from blinding of outcome assessment; (4) attrition bias arising from incomplete outcome data; (5) reporting bias arising from selective reporting; (6) other bias arising from previous interventions. Each item was rated as ‘high’, ‘low’, ‘unclear’, or ‘not applicable’ (NA) according to its potential risk of bias.

### 2.4. Data Extraction

Study information including sample size, demographics, injury characteristics, e.g., injury level, ASIA score, time since injury, setting, intervention and relevant outcome measures were extracted. Technical specifications of the end-effector devices used were extracted from manufacturer websites and compared to the operational characteristics used in the studies. These characteristics included BWS, walking speeds, step length, and cadence in lower-limb devices, and laterality, interaction, and assistance levels for upper-limb devices.

### 2.5. Data Analysis

Due to the small number of articles found and the range of outcome measures used, the results of articles of all study types were compared. Each study reported different metrics for each outcome measure (Table S2), requiring different effect size calculations to allow for comparison. All effect sizes were transformed into the r metric to allow for comparison across studies. The r metric provides a universal, bounded (−1 to 1) scale for comparison of result effect sizes independently of study sample size.

For studies providing mean difference in outcome measure results [28] or the rate-of-change in results (slope) [29], Cohen’s *d* was calculated. In both cases, due to low participant numbers, the Hedges’ *g* method was used to correct the result before the effect size and confidence interval were transformed into the *r*-metric. Where each participant’s pre- and post-intervention score was provided [30], a Wilcoxon signed-rank test was conducted. Because the study had few participants (*n* = 4), the exact *p*-values were calculated rather than normal distribution approximations. These probabilities were converted usings Fisher’s *Z* transformation, where the confidence intervals were calculated, and subsequently converted to the *r*-metric (*r* = *Z/√N*) to allow for cross-study comparisons. Other studies provided the probability results and number of participants [31,32,33]; therefore, Hedges’ *g* correction was not required, and the effect size could be calculated directly in the *r*-metric. The confidence intervals for these studies were derived using Fisher’s *Z* transformation to ensure no errors arose where confidence intervals lay near the upper and lower limits of the range (−1 to 1 for *r*).

The range of study designs, intervention durations, and participant characteristics (injury level and ASIA score), across the articles resulted in high heterogeneity. This was confirmed using Cochran’s *Q* test, which indicated moderate to substantial heterogeneity (*I*^2^ > 25%). Because of this high heterogeneity, random-effects weightings were applied when pooling effect sizes.

One RCT reported Mann–Whitney U test *p*-values, which were used to calculate effect sizes and confidence intervals because odds ratio results were only presented for some outcome measures [32]. For another RCT, the same method as described above for calculating the effect size from the mean difference (slope) was used [29]. The difference between the two groups’ Hedges’ *g* values and their standard errors was used to calculate the between-group effect sizes for each outcome measure.

### 2.6. Subgroup Analysis

Due to the small number of articles on end-effector use in both upper- and lower-limb SCI rehabilitation, the range of study designs, range of devices used, and the low level of results disaggregation by injury level or ASIA score, no subgroup analyses were performed.

## 3. Results

### 3.1. Study Characteristics

**Figure 1:**
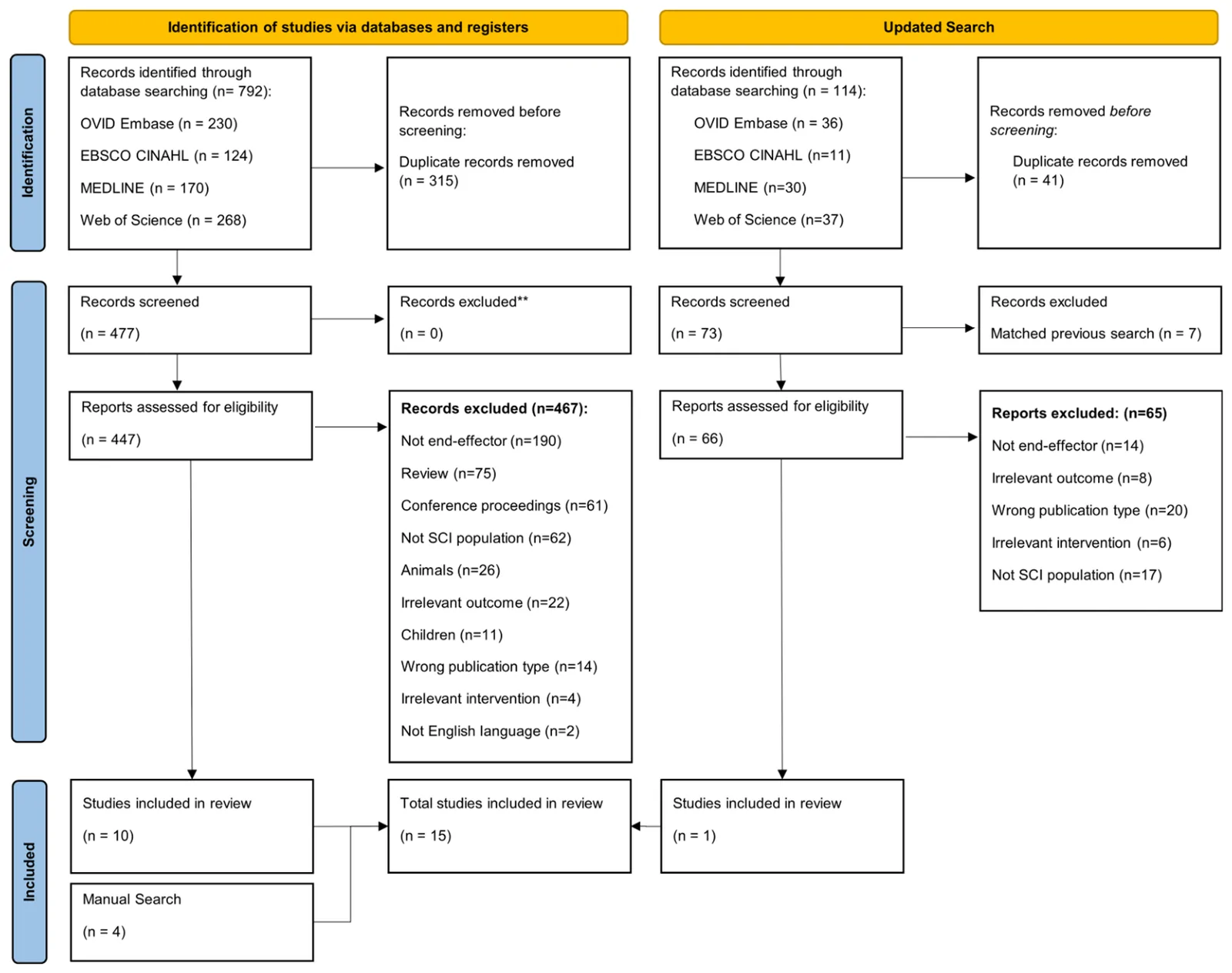
PRISMA flow diagram for the systematic search of databases and registers.

A total of 15 studies were included in the final analysis, four of which were identified through the manual search. No additional studies were identified from the manufacturers’ websites. Full details are reported in the PRISMA diagram (Figure 1).

Five studies were RCTs, whilst the remainder comprised pre–post study designs (4), cohort studies (1), prospective multi-centre studies (1), case series (1), case reports (1), comparative studies (1), repeated-measures studies (1). Further study details, including end-effector make and model and participant characteristics are listed in Table 1.

The end-effector devices included in the studies were heterogeneous. Ten studies focussed on a lower-limb end-effector, using five different devices (G-EO System [Reha Technology, Switzerland], GT I [Reha-Stim, Germany], LEXO [Tyromotion, Austria], Morning Walk [Curexo, South Korea], and LokoHelp [LokoHelp Group, Germany]). The remaining five studies on upper-limb used three commercial devices (AMADEO and DIEGO [Tyromotion, Austria], and the Haptic Master [Moog-FCS, Netherlands]).

The number of participants was also highly heterogeneous, varying from 1 (case report [34]) to a maximum of 105 participants in a RCT [29]. Excluding studies with a single SCI participant or those in which participant sex/gender was not reported, the median proportion of female participants was 35.4% (range: 14.3–60%). The mean age of participants in each study ranged from 39 [35] to 62 years old [36].

**Table 1:** Study Detail, Device Used, and Participant Characteristics.

Extremity	Author	Country	Study Design	End Effector Device	Cohort (SCI Participants only)				
					N (M, F)	Age [Years] Mean ± SD (Range)	Injury Level	ASIA	Time from Onset Mean ± SD (Range)
Lower Limb	Freivogel, Schmalohr and Mehrholz [28]	Germany	Crossover RCT	LokoHelp	1	-	T5	-	-
	Benito-Penalva, Edwards [29]	Spain	Partial RCT	End-Effector: GT I	66 (45, 21)	45	31-Tetraplegic 35-Paraplegic	A-B = 6 C = 26 D = 34	<6mo = 52 6–12mo = 5 >12mo = 9
				Exoskeleton: Lokomat	39 (26, 13)	45	14-Tetraplegic 25-Paraplegic	A-B = 5 C = 18 D = 16	<6mo = 29 6–12mo = 3 >12mo = 7
	Hesse, Werner and Bardeleben [30]	Germany	Case Series	GT I	4 (3, 1)	50 ± 8.3	1 C5/6, 1 D7/8, 1 D1/2, 1 L1/2	C = 1 D = 3	10 ± 6.3 months (3–18)
	Shin, Jeon [31]	South Korea	Prospective Multi-Centre Study	Morning Walk	13 (8, 5)	52 (19–85)	11-Tetraplegic 2-Paraplegic	C = 1 D = 12	48 Days (19–139)
	Shin, Jeon [32]	South Korea	Multi-Centre RCT	Morning Walk	16 (9, 7)	52 (32–65.2)	14-Tetraplegic 2-Paraplegic	C = 6 D = 23	54 Days (37–92)
				Conventional Therapy	13 (12, 1)	60 (55–64)	10-Tetraplegic 2-Paraplegic		44 Days (33–94)
	Calabrò, Filoni [33]	Italy	Pre-Post Study	G-EO System	15 (9, 6)	42 ± 14	2 C5, 3 C6, 1 C7, 1 T1, 1 T4, 1 T6, 3 T7, 3 T10	C = 8 D = 7	7 ± 4 months (5–14)
	Hornby, Kinnaird [35]	USA	Repeated Measure	LokoHelp	11	39 ± 15.3	1 C4–6, 1 C6, 1 C5–8, 1 C2–3, 1 T3, 1 T1, 1 T7, 1 C5, 1 T6–7, 1 C5–6, 1 C4	C = 7 D = 4	110.5 ± 120.5 Days (20–367)
	Bonnevie, Moily [37]	Australia	Comparative	LEXO	5 (2, 3)	55 (28–59)	-	-	12.5 months (6.3–18.5)
	Choi, Kim [38]	Korea	Pre-Post Study	Morning Walk	40 (21, 17)	61.8 ± 16.5	18-Tetraplegic 22-Paraplegic	-	-
	Maggio, Bonanno [39]	Italy	Cohort Study	G-EO System	42 (17, 25)	52.21 ± 15.26	-	A = 20, B = 22	7 ± 2 months
Upper Limb	Mackenzie, Tan and Benad [34]	Australia	Case Study	DIEGO	1 (M)	~50	C3	C	~10 weeks
	Kilkki, Poutanen [36]	Finland	Crossover RCT	AMADEO and DIEGO	16 (9, 7)	62 (IQR 58–66)	5 C2, 4 C3, 5 C4, 3 C5, 2 C6, 1 C8	All = D	3 years (IQR 2–7)
	Vanmulken, Spooren [40]	Netherlands	Pre-Post Study	Haptic Master	5 (4, 1)	47.2 ± 18.78	1 C5, 1 C6, 2 C7, 1 C5–7	A = 1, B = 2 C = 1, D = 1	-
	Jung, Lee [41]	South Korea	RCT	AMADEO	30 (24, 6)	47.23 ± 14.01	1 C2, 6 C3, 9 C4, 4 C5, 5 C6, 5 C7	A = 3, B = 4 C = 7, D = 16	7.53 ± 6.51 months
				Occupational Therapy		53 ± 13.5			7.62 ± 5.22 months
	Mackenzie, Tan and Benad [42]	Australia	Pre-Post Study	DIEGO	7 (6, 1)	44.29 (95% CI: 30.96–57.61)	7 C4	B = 4 C = 2 D = 1	-

IQR—Inter-quartile Range.

Participants’ level of SCI injury varied across studies, with some studies reporting tetraplegic or paraplegic. Studies included a high proportion of participants with C and D ASIA scores, 25% and 48%, respectively, whereas only 27% had ASIA scores of A or B. In addition, time since onset ranged from approximately 1 month [31] to 7 years [36].

One RCT disaggregated the results by ASIA score, injury level, etc. [29]; however, these results contained both the end-effector and control groups results. Another separated the participants into those with improved 10-m Walk Test (10MWT) results and those whose results had not improved, with numbers of participants, their characteristics, and other outcome measure results reported for each group [32]. The remaining three RCTs did not disaggregate the results by any participant characteristics.

### 3.2. Quality Assessment

Of the 15 studies, 14 showed an overall high risk of bias and one was rated as unclear (Table 2). In non-RCT studies, the most frequent limitations were related to blinding of participants and personnel and blinding of outcome assessment. In addition, none of the studies reported whether participants had received previous interventions, resulting in an unclear score. By contrast, some domains were consistently strong. For example, incomplete outcome data and selective reporting were rated as having a low risk of bias in 13 and 14 studies, respectively.

### 3.3. Therapeutic Protocols

#### 3.3.1. Lower-Limb Study Protocols

Study durations ranged from single-session feasibility studies [35,37] to intensive multi-week programs lasting from 3 to 8 weeks [28,29,30,31,32,33,38] and up to six months in longitudinal rehabilitation protocols [39]. Session frequencies typically ranged from three to six sessions per week, with sessions lasting 30–60 min (Table S1).

Treatment protocols frequently combined robotic training with conventional physiotherapy, occupational therapy, and psychological support, promoting active engagement and multi-modal rehabilitation (Table S1). A few interventions also integrated FES [30] or visual feedback [37], whereas others provided minimal setup details.

#### 3.3.2. Upper-Limb Study Protocols

Study durations ranged from three [34] to six weeks [36,40], with all studies taking place in a clinical setting (hospital or community/rehabilitation centre). Session frequency ranged from three 40-min sessions [41] to six 1-h sessions [34] a week. One study conducted two 30-min sessions, separated by a 30-min break [40].

Treatment protocols were often conducted alongside occupational therapy [34,41,42], with one study specifying the functional tasks completed during the end-effector rehabilitation sessions [34] and another leaving the tasks and device choice up to the patient [36].

### 3.4. Device Characteristics

All eight devices used in the studies included in this review were commercially available (Table 3 and Table 4). All the lower-limb devices (Table 3) are classified as having 2 to 3 degrees of freedom of movement for each footplate and belong to the gait-training rather than the balance-training category of end-effector rehabilitation devices [17].

The ‘Device Technical Specifications’ described (Section 3.4.1, Table 3 and Table 4) were obtained from website sources for the commercially available devices, whereas the ‘Device Settings Used’ (Section 3.4.2, Table 3 and Table 4) refer to the settings described in articles included in this review. Walking speed units varied, with commercial device websites often using kilometres per hour (km/h) and research articles using either km/h or metres per second (m/s). Throughout this section and in Table 3, both units are reported to increase ease of comparison. All step lengths are reported in millimetres.

#### 3.4.1. Device Technical Specifications

##### Lower-Limb Devices

The available BWS is advertised as up to 180 kg for the LEXO device and up to 200 kg for the Morning Walk, G-EO, and GT-II devices (Table 3). BWS is not provided directly with the LokoHelp end-effector device; however, it can be used in conjunction with BWS treadmills.

The walking speeds of the commercial devices are listed as having maximum speeds ranging from 2 km/h (0.56 m/s) (GT-II and LokoHelp) to 3.3 km/h (0.92 m/s) (Morning Walk) (Table 3). The G-EO device can set its cadence and step length from zero up to the maxima of 70 steps/min and a 550 mm step length, respectively, whereas the Morning Walk and GT-II devices have minimum step lengths of 300 mm and 340 mm, respectively. The LokoHelp has a fixed step length due to its design; however, it can be purchased in two sizes with 300 mm or 400 mm step lengths [43].

##### Upper-Limb Devices

Three upper-limb end-effector devices (AMADEO, DIEGO, and Haptic Master) were used across the five articles. Two of the devices were designed for whole-arm movements, supporting the rehabilitation of shoulder and elbow movement, while the third was designed solely for hand/finger rehabilitation (Table 4). Unlike in lower-limb rehabilitation, where the cyclical movements of walking are the main priority in rehabilitating mobility, whole-arm movements in upper-limb rehabilitation are much more varied and task-specific. The two devices which aid with whole-arm rehabilitation, DIEGO and the Haptic Master, both attach to the user using a support on the forearm as the end-effector [40,44]. The AMADEO device, which focuses on hand/finger rehabilitation, restricts arm movement at the wrist and uses end-effectors on the fingertips to generate movement of the fingers [45]. Both devices by Tyromotion offer active, passive, and assistive training modes, with gamification available on a screen to aid in rehabilitation enjoyment [44,45].

**Table 3:** Lower-Limb End-Effector Gait Trainer Technical Specifications and Operational Characteristics.

Lower-Limb Devices	Device Technical Specifications				Device Operational Settings Used			
	Body Weight Support	Walking Speed	Cadence (Steps/ min)	Step Length (mm)	Body Weight Support	Walking Speed	Cadence (Steps/min)	Step Length (mm)
LEXO	Harness or Saddle ≤180 kg [46]	Step length, cadence, and walking speed are fully programable [46]			Median (IQR) 8% (34–2%) [37]	-	IQR 292-2062 steps/Session (7–27 min) [37]	-
G-EO	Harness, ≤200 kg 0–5 cm vertical movement [47]	≤2.3 km/h [47] (6.4 m/s)	0–70 [47]	0–550 (85–220 step height) [47]	80–10% [33]	-	-	-
GT I	Harness [30] ≤ 200 kg * [48]	≤2 km/h [48] (0.56 m/s)	-	340–480 * [48]	<40% [29] 35–10% [30]	>1.5 km/h [29] (0.42 m/s) 1–2 km/h [30] (0.28–0.56 m/s)	800–1250 steps/session (20–25 min) [30]	430–490 [30]
		60–40% stance/swing ratio [30]						
Morning Walk	Saddle ≤ 200 kg 15–80% [49]	≤3.3 km/h [49] (0.92 m/s)	4–75 [49]	300–730 *** [49]	20% [31,32]	** 0.54 km/h (0.15 m/s) [31,32]	>30 [31,32]	>300 [31,32]
LokoHelp	-	≤2 km/h [43] (≤0.56 m/s)	-	Fixed at 300 or 400 [43]	30–10% [28] 40% [35]	≤2.5 km/h [28] (0.69 m/s) 1.5 km/h [35] (0.42 m/s)	44 [35]	400 [28] 500 [35]

* Newer version, GT-II available online with technical specifications presented above. ** Estimated value from reported cadence and step length. *** Webpage translated by Microsoft Edge. Page described the metric as stride length; however, compared to competitor devices and articles using the device this could be step length.

**Table 4:** Upper-Limb End-Effector Device Technical Specifications and Operational Characteristics.

		Upper-Limb Devices		
		DIEGO	Haptic Master	AMADEO
**Device** **Technical** **Specification**	**Joints Mobilised**	Proximal [44]	Proximal [40]	Distal [45]
	**Laterality**	Uni- and Bi-lateral [44]	Unilateral [40]	Unilateral [45]
	**Assistance**	Active, Passive, Assistive [44]	Passive, Active, and Active-Assisted [40]	Active, Passive, Assistive [45]
	**Interaction**	Gamification, VR, Functional Tasks [44]	-	Gamification [45]
	**Feedback**	Yes	No	Yes
**Device** **Operational** **Settings Used**	**Laterality**	Bilateral [34,36,42]	Unilateral [40]	Unilateral [36,41]
	**Weight Support**	Gravity Free [34] Weight Relief [36]	-	N/A
	**Assistance**	-	Passive, Active, and Active-Assisted [40]	Passive and Assistive [41]
	**Interaction**	Gamification and Functional Tasks [34,42]	Functional Tasks [40]	Gamification [41]

#### 3.4.2. Device Operational Settings Used

##### Lower-Limb Articles

Only one article used the LEXO end-effector device, in which the device settings were left to the participant and physiotherapist to agree on. These included the percentage of BWS, passive or active modes, walking parameters, and duration [37]. The chosen operational use (gait kinematic) settings for each participant are not reported; therefore, Table 3 does not include the walking speed and step length data for this device.

Similarly, the two articles that used the G-EO device used personalised patient centred rehabilitation programs that were adaptable throughout the intervention period [33,39], meaning the gait parameters used and how they changed throughout are not reported. Calabrò, Filoni [33] describe how the 10MWT was used to determine the mean gait parameters used, with the walking speed set at 0.4 m/s (1.44 km/h) and increased by 0.5 m/s (1.8 km/h) every three minutes until the maximum tolerable walking speed was determined, subsequently the session then began. Furthermore, BWS started at 80% in week one and was reduced by 10% each week whenever tolerable [33]. However, the progression of walking speed, BWS, and gait parameters for each participant throughout the intervention is not reported.

Hesse, Werner and Bardeleben [30] used a custom device developed by their university (Free University Berlin) in collaboration with the University of Teesside, UK [50], which later formed the spin out company Reha-Stim and their first commercially available gait trainer device, the GT-I [51]. Benito-Penalva, Edwards [29] also used this GT-I device. Their reported gait training program states how participants (*n* = 66) started with the end-effector device at 40% BWS and 1.5 km/h (0.42 m/s) walking speed, with each session reducing the BWS and increasing walking speed where tolerable [29]. The cadence and step lengths are not discussed in the article. Hesse, Werner and Bardeleben [30] conducted a case series of four participants, reporting individually how each case used the device across a 5-week rehabilitation program. All four participants increased their walking speed and reduced their BWS throughout the intervention period, with participants one and three also increasing their step length and the duration between rest periods.

Two of the three articles which used the Morning Walk device were from the same research group and used the same approach to setting the end-effector parameters. Each participant began the session with BWS at 20%, a 300 mm step length (device minimum), and a 30 steps/min cadence [31,32]. Together, these equate to a walking speed of 0.15 m/s (0.04 m/s) (Table 3). Each parameter was adjusted relative to participant performance, with cadence increased by 5 steps/min if a 10-min session was completed without rest. Progression of these parameters throughout the intervention for each participant was not presented or discussed. The third article by Choi, Kim [38] included participants who could use the end-effector device for a 5-min flat walking session and grouped them based off their required BWS (>70%, >60%, and <60%). However, the stride lengths, cadence, and walking speeds used throughout the intervention period are not presented or discussed.

Finally, two studies used the LokoHelp device [28,35]. Hornby, Kinnaird [35] discuss the use of the device for a single session only, where they fixed the walking speed at 1.5 km/h (0.42 m/s) with a step length of 500 mm (N.B. this differs from the currently available device step lengths displayed in Table 3). Freivogel, Schmalohr and Mehrholz [28] used the 400 mm step length on the LokoHelp device for a 6-week intervention. The walking speeds were individually set between zero and 2.5 km/h (0.69 m/s), with the initial BWS set between 10% and 30%, which was reduced whenever possible [28]. The walking speeds used were not documented throughout the intervention; however, the mean and standard deviation for distance walked within the 30-min sessions is reported as 553 ± 116 m. This distance walked equates to a mean cadence of 46 steps/min and a mean walking speed of 0.31 m/s, assuming no rest periods.

##### Upper-Limb Articles

Two articles by the same research group used the DIEGO device, with the first study being a case report [34] and the second including more participants (*n* = 7) in a pre-post study design [42]. The case report used functional tasks and gamification with a focus on sagittal plane movements [34]. In their pre-post study, 30 min of games were completed followed by 20 min of functional tasks in each session. The games/tasks completed were recorded in a diary, with repetitions and difficulties noted and adjusted throughout the intervention [42]. The progression of activities recorded in the diary is not presented; instead, the article focuses on both participants’ and clinicians’ personal experiences of using the DIEGO device. However, the participant quotes discuss how the device and gamification increased the drive and motivation to train but do not overcome the monotony of daily repeated rehabilitation [42].

Vanmulken, Spooren [40] used the Haptic Master device to assist the participants whilst completing a range of functional tasks such as eating with cutlery, using a purse, and moving a cup. The tasks were gradually increased in difficulty using segmentation and the T-TOAT method [52]. This scalable approach to functional activities was enabled through the progression of the device’s passive, active-assisted, and active modes, which gradually increased the work required by the user through reducing the assistance provided by the device.

Jung, Lee [41] used the AMADEO device, which, unlike the whole-arm devices described previously, provides very constrained and linear motion paths for the end-effectors as they are attached to the fingertips and only generate finger flexion and extension movements [45]. In their study, the therapist chose different available training programs on the device depending on the participants’ specific difficulties and weaknesses.

Finally, in the remaining upper-limb article, Kilkki, Poutanen [36] used multiple devices in their study. The three assisted devices used were the DIEGO, PABLO, and AMADEO devices; however, in this review, the PABLO device is not discussed as it is not an end-effector device. The devices used, task-specific training, and device modes were all controlled by the therapist, with the total time on each device reported for each participant (*n* = 16).

### 3.5. Outcome Measures Used

The functional outcome measures used are detailed with their results in Table S2 (lower-limb) and Tables S3–S5 (upper-limb). The outcome measures most commonly assessed in lower-limb studies were walking speed with the 10MWT (six studies); functional ambulation with the Walking Index for Spinal Cord Injury [WISCI II] (five studies); lower-limb strength and motor recovery with the Lower Extremity Motor Score [LEMS] (four studies); walking endurance with the 6-Minute Walk Test [6mWT] (three studies); Berg Balance Scale [BBS] (three studies); Functional Ambulation Category [FAC] (two studies); lower-limb strength with the Modified Ashworth Scale [MAS] (two studies); Spinal Cord Independence Measure [SCIM-III] (two studies); and proprioception [ISNCSCI] (two studies).

Additional outcome measures used by single articles were Fugl-Meyer [39]; centre of pressure [30]; PASIPD, FES-I, Borg Rating Scale of Perceived Exertion, overall fatigue and enjoyment [37]; Medical Research Council scales of the lower extremities [38]; Rivermead Mobility Index, distance walked during training sessions, discomfort of patients and therapists, and physical stress of patients and therapists [28]; and GaitMat II (EQ Inc, Chalfont, Pennsylvania) preferred overground gait speed, and timing of unilateral stance and swing gait phases [35].

Upper-limb studies [34,36,40,41,42] assessed strength (five studies), independence with the SCIM-III (five studies), range of motion (two studies), Upper Extremity Motor Score [UEMS] (two studies), pain (two studies), fatigue (two studies), and functional performance using validated clinical tools (e.g., COPM, two studies; GRASSP, one study; and rehabilitation goals [International Classification of Functioning, Disability and Health codes] scored using the Goal Attainment Scale T score, one study).

Other non-functional outcome measures used included Beck Depression Inventory [33,39], Montreal Cognitive Assessment [39], Short Form of the Patient Satisfaction Questionnaire [39], Short Form-12 Health Survey (SF-12) [39], Short Form (SF-36) health survey [33], European Quality of Life Scale [37], Canadian Occupational Performance Measure (COPM) [34], Usefulness Satisfaction and Ease-of-use questionnaire [40], and the intrinsic motivation inventory [40].

#### 3.5.1. Lower-Limb Outcome Measure Results

**Figure 2:**
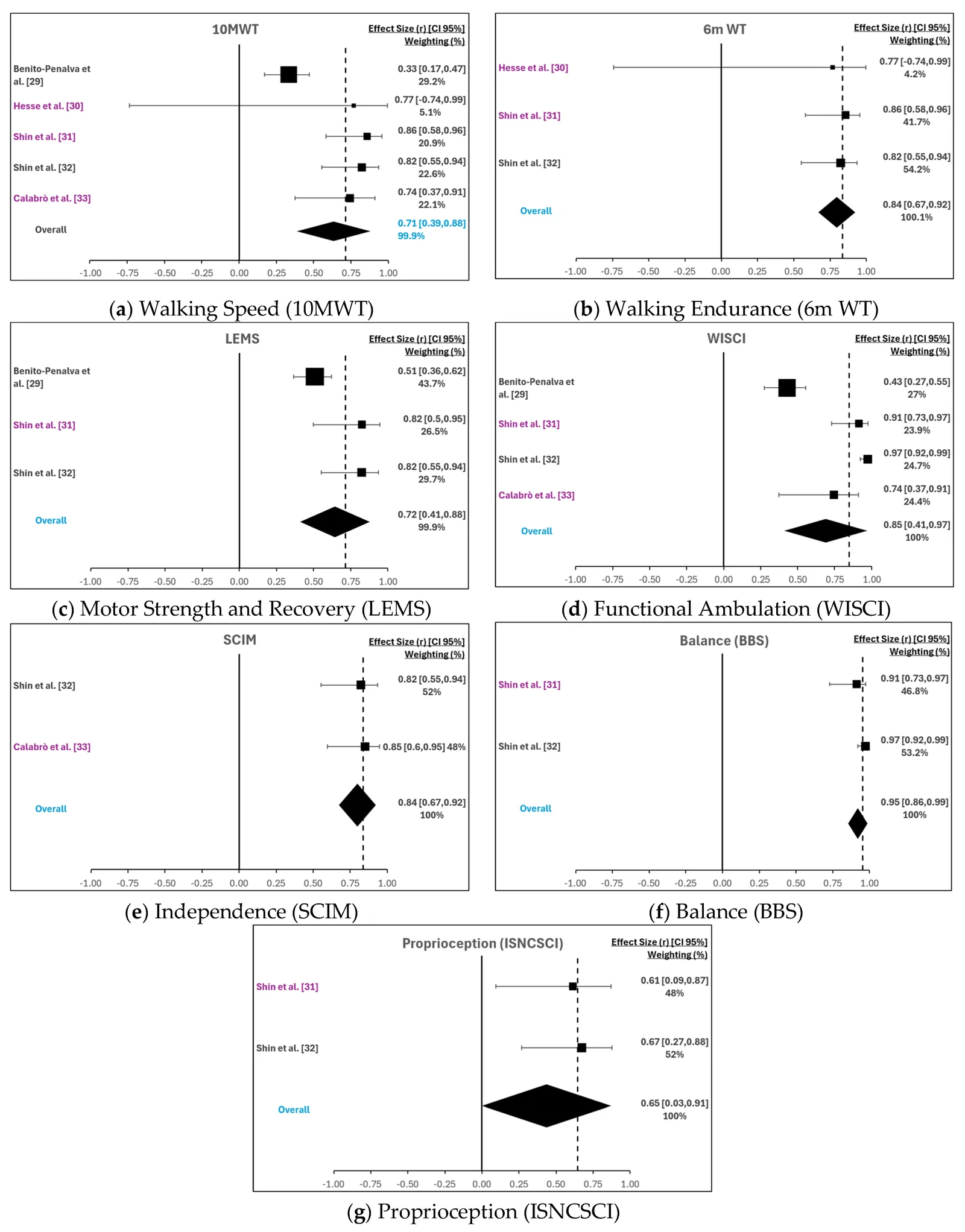
Forest plots for pre- to post- gait-trainer intervention changes in lower-limb outcome measure results. X-axes display the linear scale effect size result (Negative = reduction in outcome measure, Positive = improvement in outcome measure), with the Null line at 0. Studies included in each graph are detailed down the left-hand side ((**a**) [29,30,31,32,33], (**b**) [30,31,32], (**c**) [29,31,32], (**d**) [29,31,32,33], (**e**) [32,33], (**f**) [31,32], and (**g**) [31,32]). RCTs study authors are written in black, non-randomised studies in purple, and the overall in blue. The result values of effect size, confidence interval range, and weighting towards overall result are included down the right-hand side.

**Figure 3:**
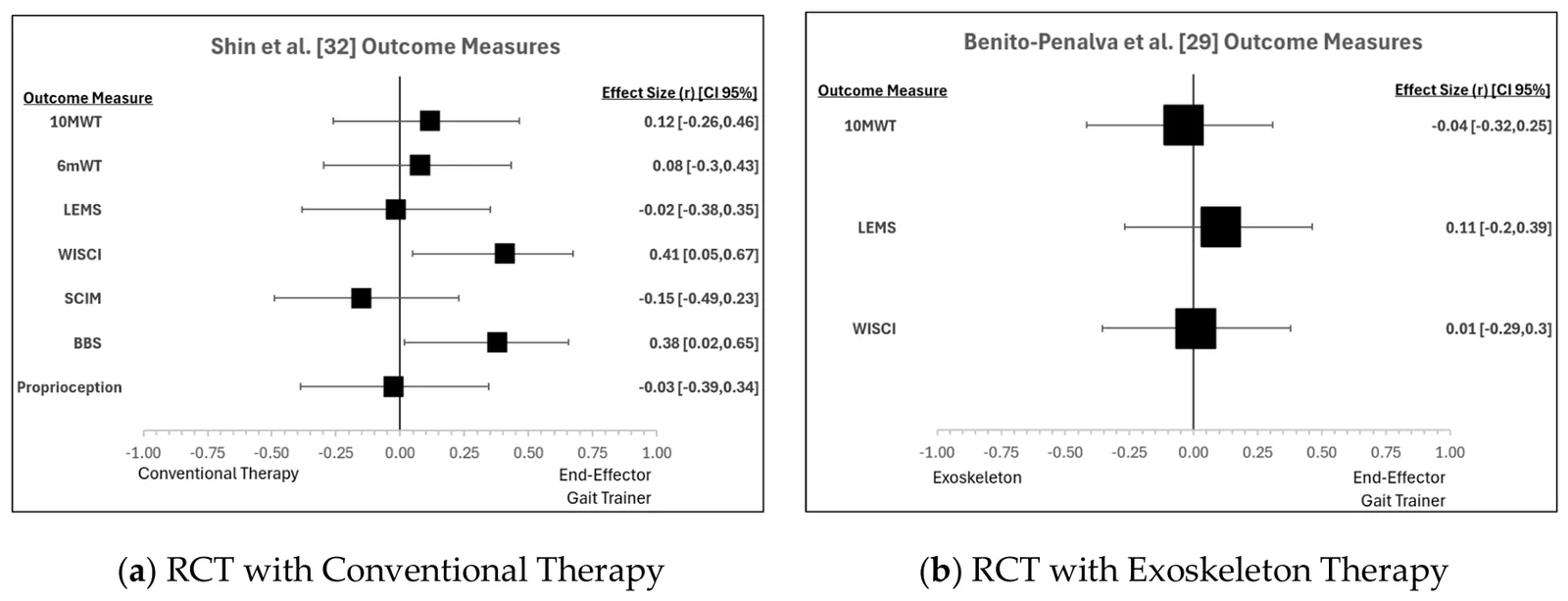
Forest plots comparing the two groups used in the two lower limb randomised controlled trial articles. X-axes display the linear scale effect size result (Positive = greater improvement in end-effector group Negative = greater improvement in compared intervention) with the Null line at 0. (**a**) The outcome measures used by Shin et al. [32] comparing an end-effector gait trainer to conventional therapy. (**b**) The outcome measures used by Benito-Penalva et al. [29] comparing an end-effector gait trainer to an exoskeleton gait trainer.

The forest plots in Figure 2 show the changes in outcome measures where more than one article reported the same measure. Due to the different study designs, these plots show the pre–post change scores for each article and outcome measure, expressed using linear-scale effect size metrics. Additionally, two studies were RCTs which compared end-effector gait trainer interventions to exoskeleton [29] and conventional therapy [32]; these comparisons are shown in Figure 3. As both Figure 3a,b contain only a single study, each study’s individual outcome measures are shown with no overall effect size. The articles included in this review not shown in Figure 2 or Figure 3 can be seen in Table S2. These articles either used different outcome measures to all other articles or did not disclose or disaggregate results for their SCI participants sufficiently enough to be analysed.

The overall combined effect size and confidence interval ranges for each metric suggest that the use of an end-effector gait trainer has a medium-to-large effect on improving the results in each outcome measure. Balance, SCIM, WISCI, and the 6mWT all had very large effect sizes of >0.8. The proprioception outcome measure had the lowest overall effect size (r = 0.65) and the largest confidence interval range (0.03–0.91), suggesting that there is still some uncertainty about the influence end-effector use has on this outcome for different individuals; however, only two studies included this outcome measure (Figure 2g). Caution should be taken when interpreting these overall effect size results due to high risk of bias in all studies (Table 2) and the small number of studies (n = 2 to 5) included for each outcome measure, of which only one or two were RCTs.

Only one study, Hesse, Werner and Bardeleben [30], produced confidence interval ranges large enough to suggest that end-effector gait training could have no effect on the outcome measures recorded for some individuals (shown on the 10MWT (Figure 2a) and 6mWT (Figure 2b) forest plots). The large confidence interval is due to the very small number of participants in this case series, which is also reflected in the low weightings assigned to this study when calculating the overall effect size results.

The RCT by Shin, Jeon [32] shows that end-effector gait trainer use in rehabilitation for people with spinal cord injuries has a moderate effect on improving outcomes in both balance (BBS) and functional ambulation (WISCI) in comparison to conventional therapy rehabilitation. Results for all other outcome measures were comparable and therefore suggest either no or only a trivial effect in favour of either method (Figure 3a). The partial-RCT by Benito-Penalva, Edwards [29] shows that end-effector and exoskeleton gait trainers perform comparably when assessed using the 10MWT, LEMS, and WISCI outcome measures (Figure 3b).

#### 3.5.2. Upper-Limb Outcome Measure Results

Upper-limb studies demonstrated some consistent reporting, with all studies assessing independence with the SCIM-III; however, some focused on specific subsections of this tool. Conversely, considerable variability in the outcome measurement tools used can be seen across strength, range of motion, pain, fatigue, and functional performance (e.g., COPM, GRASSP). In addition, a wide range of patient-reported and non-functional outcomes were included, such as measures of mood, cognitive function, quality of life, and user experience and motivation.

This diversity in outcome selection reflects a lack of consensus regarding the primary therapeutic targets of upper-limb end-effector interventions, with studies variably prioritising impairment-level, functional, and psychosocial outcomes. As a result, direct comparison of intervention effects across studies is limited, and it remains unclear which domains are most responsive to end-effector rehabilitation. This heterogeneity also suggests that upper limb interventions may address broader aspects of recovery beyond motor function alone, but at the expense of consistency in outcome reporting.

## 4. Discussion

The low number of articles found in this review that matched the inclusion criteria, combined with the wide range of study designs seen across the articles, suggests that there is a strong need for more studies investigating end-effector use specifically in populations with SCI. Only four studies included in this review were classified as RCTs, totalling 165 participants across them, whereas a similar review focussing on end-effector use and stroke patients found nine RCT’s, totalling 591 participants [53]. Furthermore, the disaggregation of results by injury level, ASIA score, etc., was inconsistent, making investigations into how these characteristics influence participant outcomes not possible.

Across all studies, the risk of bias associated with blinding of personnel was rated as high, which is mainly due to the nature of the rehabilitation devices being used and cannot be avoided (i.e., patients are fully aware of whether they are using an end-effector device in rehabilitation or not). Additionally, the range of study designs included contributes to the high risk of bias results as non-randomised protocols are inherently prone to increased bias [27]. Therefore, as previously mentioned, caution should be taken when interpreting the results of these articles.

The RCT by Benito-Penalva, Edwards [29] demonstrated the smallest effect size among the studies presented in Figure 2, despite an intervention duration of eight weeks, comparable to or longer than that of other included studies. One potential explanation is the inclusion of conventional therapy (CT) alongside end-effector gait training, for which limited detail was provided. The addition of CT in other studies may have introduced variability in motor task practice, such as activities of daily living, potentially promoting broader functional adaptations (e.g., strength gains) that translated into improvements in specific gait outcome measures (e.g., 10MWT, 6mWT). Task variability is suggested to promote the development of diverse motoneuronal network patterns, with interactions between these networks potentially enhancing motor recovery [54]. However, this must be balanced against the need for task specificity, as excessive variability may dilute targeted training effects.

Furthermore, the RCT by Benito-Penalva, Edwards [29] had the largest number of participants across all studies reviewed in this paper and included participants with ASIA levels of A through D, with other studies only including ASIA levels C and D [30,31,32,33,35]. This increases the representation of the SCI patient population demonstrates how the impairment level of a patient could influence the effect of the gait therapy they receive. Regardless, the positive effect size and narrow confidence interval show that end-effector gait training can improve mobility outcomes across all ASIA impairment levels.

All of the lower-limb devices described in the articles included in this review were end-effector gait trainers designed for gait rehabilitation only and not balance (as categorised by Diego, Herrero [17]). The level of detail reported on the gait characteristics used by the participants also varied between studies. Some studies described some structure around how the gait characteristics were chosen, such as starting at a specific cadence, step length, and BWS, and changing them once the session could be completed, whereas others left the gait characteristics up to the decision of the physiotherapist and participant. However, the 10MWT and WISCI-II are the most reliable and clinically useful measures [55], which were the two most common outcome measures used across the lower-limb articles in this review, representing 60% and 50% of articles, respectively (Table S2). Similarly, the articles which focused on whole-arm upper-limb rehabilitation did not report which of the available task/training exercises the participants completed or used. One study mentioned a focus on sagittal plane movements [34]; however, this may have suited the case study participant’s requirements.

The study by Freivogel, Schmalohr and Mehrholz [28] compared conventional gait therapy to end-effector gait trainer therapy. The results are not displayed in Figure 2 and Figure 3 because the study included only a single SCI participant; however, the study used some additional outcome measures to display the benefits of end-effector systems. The first benefit is that using end-effector gait trainers removes the requirement of a second therapist to help with the therapy session. Secondly, they measured the total distance walked during the training sessions and found that an additional 153 m (38.25%) was walked on average in the end-effector training sessions [28]. A study into end-effector exercise intensity also used step count as an outcome measure, finding a median of 1964 steps over a median exercise duration of 20 min [37]. In the case series reported by Hesse, Werner and Bardeleben [30], they aimed for 1250 steps/session and achieved between 800 and 1200 steps in the first therapy session. Repetition and intensity are key principles of neuroplasticity and aid in the rehabilitation of motoneuronal pathways. An increase in rehabilitation intensity has been shown to increase muscle activity and improve spatiotemporal gait parameters towards normal healthy values [56]. However, in upper-limb rehabilitation, repetition reporting is complicated further as the tasks can be less cyclical than in lower-limb rehabilitation. Kilkki, Poutanen [36] included multiple devices in their upper-limb study, in which they presented the total time each participant completed on each device over the intervention, highlighting which participants focused more on proximal or distal functional movements.

The range of end-effector walking speeds reported by each article (0.15 m/s [31,32] to ≤0.69 m/s [28]) spans multiple functional ambulation levels [57]. This indicates that participants with markedly different mobility capacities were trained under broadly similar protocols, raising concerns regarding the comparability of intervention dosing across studies. Such variability likely contributes to the heterogeneity in reported outcomes and complicates the identification of optimal training parameters.

In contrast to lower-limb rehabilitation, which is characterised by cyclical and rhythmic movement patterns such as gait, upper-limb function is inherently more discrete and task-dependent, involving actions such as reaching, grasping, and object manipulation. This fundamental difference has important implications for outcome assessment and interpretation. While lower-limb interventions can be evaluated using relatively standardised, performance-based measures (e.g., walking speed), upper-limb studies often employ a wider range of outcomes spanning impairment, functional performance, and psychosocial domains. This variability reflects the absence of a single defining functional task for the upper limb and contributes to inconsistency in reported effects across studies. Moreover, the discrete and context-dependent nature of upper-limb movements means that improvements may be task-specific and not readily generalisable across outcome measures. This is highlighted by the SCIM-III, a reliable and valid outcome measure [58], being used in all studies, although some only reported results for specific subsections. Consequently, linking intervention parameters to clinical outcomes is more challenging in upper-limb rehabilitation, as observed effects are likely influenced not only by the intervention itself but also by task selection, measurement approach, and individual movement strategies.

The authors of this review acknowledge that reporting end-effector operational use characteristics for each participant and each session throughout an intervention would generate a large and difficult dataset to record and present. However, without this information, it is impossible to analyse how device operational use characteristics (kinematic quality of movements) may influence the outcomes of the participants. Individualisation is a key advantage of end-effector gait trainers, as they can easily be adjusted throughout a session and intervention to meet users’ needs. Therefore, to enable future studies to report this information in detail easily and consistently, a suggested reporting metric should be developed.

Current standardised frameworks, such as the TiDieR-Rehab Checklist [59], provide guidance on how rehabilitation interventions should be reported in literature. For end-effector robotic rehabilitation, two sections of the TiDieR-Rehab Checklist refer to the device operational characteristics used: Section 9—How Much (intervention length, frequency of session, session length, and session elements); and Section 11—Regression/ Progression (changes to the amount or challenge level of rehabilitation) [59]. However, the session elements include time and repetitions of active participation within a session, which, as already discussed, are not well reported throughout an intervention. Furthermore, the kinematic quality of movements can still vary within these reported metrics, which is the gap in rehabilitation reporting highlighted by this review. To enable data collection of this type, along with improved TiDieR reporting without undue demand on clinicians, device interfaces and reporting metrics require clinical input, as systems which are either over- or under-constrained can complicate their use and be detrimental to clinical use [60], or compromise the patient–clinician relationship, leading to demotivation of patients [61]. In lower-limb rehabilitation, whole-session measurements of total distance walked, steps completed, and total rest time could provide an indication into the level of ‘intensity’ and ‘repetition’ for a particular participant throughout a session of a specified duration. Simple reported metrics such as these could help provide more insight into end-effector operational use and how it may influence the rehabilitation of people with SCI.

### Limitations

In this review, only a small number of articles were analysed. Moreover, the range of study designs (mostly non-randomised); intervention task, frequency, and duration; inclusion of simultaneous interventions; participant SCI level and maturity; and ASIA level increased the degree of variability seen within the results across the articles reviewed. This is further amplified by the inherently discrete and task-dependent nature of upper-limb function, as well as the diversity of outcome measures employed. Differences in task selection, device capabilities, and participant characteristics make comparison across studies particularly challenging, limiting the ability to draw clear conclusions regarding intervention effectiveness. These findings emphasise the need for more rigorous study designs and greater standardisation in both intervention protocols and outcome reporting to better understand the clinical utility of upper-limb end-effector interventions.

## 5. Conclusions

In pre-post intervention analysis, rehabilitation including end-effectors is shown to have a positive influence on functional outcomes of people with SCI, with ASIA impairment level potentially influencing this effect strength. Reporting of clinically validated outcome measures was high, with 60% and 50% of lower-limb studies using the 10MWT and WISCI-II, respectively, and all upper-limb studies using an aspect of the SCIM-III. Nevertheless, reporting of end-effector gait trainer operational use (kinematic) settings used throughout the therapeutic sessions lacks detail across the articles found in this review. The operational use settings were often described as initial settings, targets, or left to the expertise of the physiotherapist, with the settings adapted in each session to suit the participants. The adaptation of these settings both within and between sessions is important for patient rehabilitation, making them difficult to report; however, reporting whole-session metrics such as the number of steps, total distance walked, and rest periods taken for each session reduces the complexity of reporting and would allow for a more in-depth analysis of how the time-varying kinematics used may be influencing the efficacy of the intervention.

## Figures and Tables

**Table 2:** Risk of Bias Quality Assessment Results for Included Studies.

	Freivogel, Schmalohr and Mehrholz [28]	Benito-Penalva, Edwards [29]	Hesse, Werner and Bardeleben [30]	Shin, Jeon [31]	Shin, Jeon [32]	Calabrò, Filoni [33]	Mackenzie, Tan and Benad [34]	Hornby, Kinnaird [35]	Kilkki, Poutanen [36]	Bonnevie, Moily [37]	Choi, Kim [38]	Maggio, Bonanno [39]	Vanmulken, Spooren [40]	Jung, Lee [41]	Mackenzie, Tan and Benad [42]
Type of Study Design	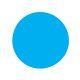	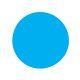	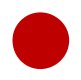	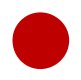	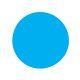	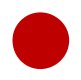	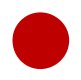	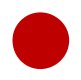	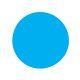	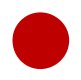	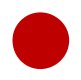	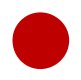	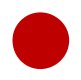	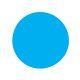	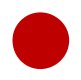
Random Sequence Generation ^1^	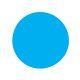	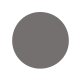	N/A	N/A	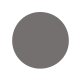	N/A	N/A	N/A	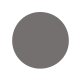	N/A	N/A	N/A	N/A	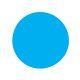	N/A
Allocation Concealment ^1^	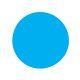	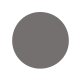	N/A	N/A	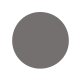	N/A	N/A	N/A	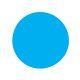	N/A	N/A	N/A	N/A	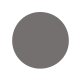	N/A
Blinding of Participants and Personnel ^2^	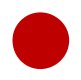	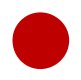	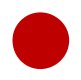	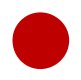	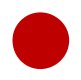	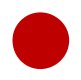	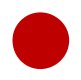	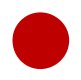	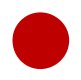	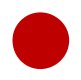	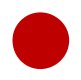	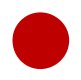	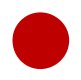	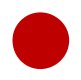	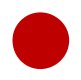
Concurrent Intervention/Unintended Exposure	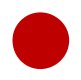	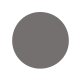	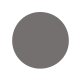	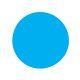	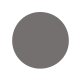	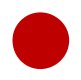	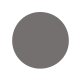	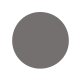	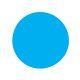	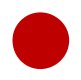	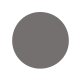	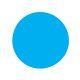	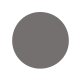	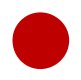	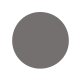
Blinding of Outcome Assessment ^2^	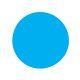	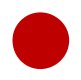	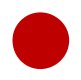	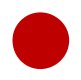	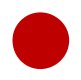	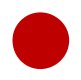	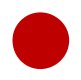	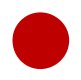	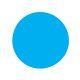	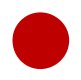	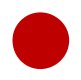	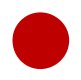	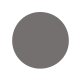	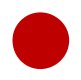	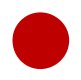
Incomplete Outcome Data ^2^	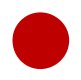	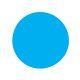	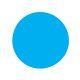	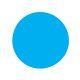	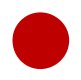	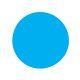	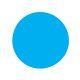	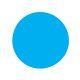	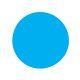	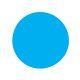	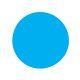	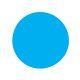	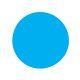	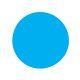	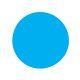
Selective Reporting	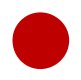	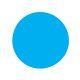	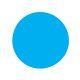	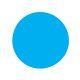	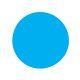	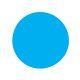	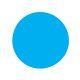	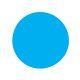	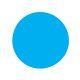	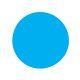	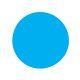	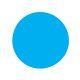	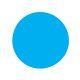	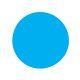	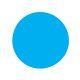
Previous Interventions	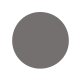	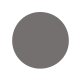	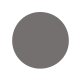	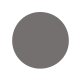	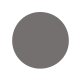	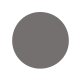	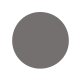	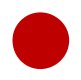	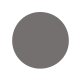	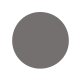	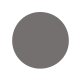	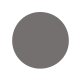	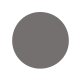	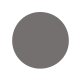	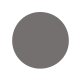
Summary Assessment ^2^	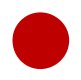	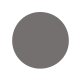	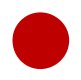	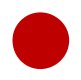	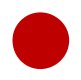	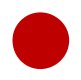	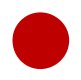	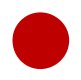	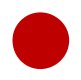	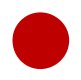	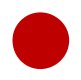	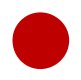	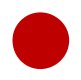	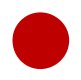	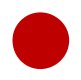

^1^ Only applicable to randomised controlled trials. ^2^ Assessments should be made for upper- and lower-limb functional outcome measures. Results presented as; Red-High, Blue-Low, Grey-Unclear.

## Data Availability

No new data were created or analyzed in this study. Data sharing is not applicable to this article.

## Supplementary Materials

The following supporting information can be downloaded at: https://www.mdpi.com/article/10.3390/bioengineering13101138/s1, Section S1: Search Terms; Table S1: Details of Study Intervention Protocols; Table S2: Lower-Limb Functional Outcome Measures and Results; Table S3: Upper-limb Functional Outcome Measures and Results; Table S4: Upper-limb Strength Outcome Measures and Results; Table S5: Upper-limb Range of Motion Outcome Measures and Results.

## Abbreviations

The following abbreviations are used in this manuscript:

SCI	Spinal Cord Injury
ASIA	American Spinal Injuries Association Impairment Scale
BWS	Bodyweight Support
PRISMA	Preferred Reporting Items for Systematic Reviews and Meta-Analyses
RCT	Randomised Controlled Trial
FES	Functional Electrical Stimulation
IQR	Inter-quartile Range
10MWT	10-Meter Walk Test
km/h	Kilometres per Hour
m/s	Metres per Second
WISCI	Walking Index for Spinal Cord Injury
LEMS	Lower-Extremity Motor Score
6mWT	6-Minute Walk Test
BBS	Berg Balance Scale
FAC	Functional Ambulation Category
MAS	Modified Ashworth Scale
SCIM	Spinal Cord Independence Measure
UEMS	Upper-Extremity Motor Score
COPM	Canadian Occupational Performance Measure
CT	Conventional Therapy
