# Supplementary material for: End-Effector Robotic Rehabilitation After Spinal Cord Injury: A Systematic Review of Devices, Operational Characteristics Used, Outcome Measures, and Rehabilitation Effectiveness
Source: Bioengineering (Basel). 2026 Sep 29;13(10):1138. doi: 10.3390/bioengineering13101138 (PMC13701345; doi:10.3390/bioengineering13101138)
Supplement: Supplementary file 1 [file bioengineering-13-01138-s001.zip › bioengineering-4517232-supplementary.pdf]

S. Supplementary Material

S.1. Search Terms

S.1.1. Web of Science

(TS=('Spinal Cord injur*' or ‘Spinal Cord contusion*' or Spinal Cord trauma*' or Spinal Cord transection*' or Spinal Cord laceration*' or Spinal Cord compression*' or Spinal Cord damag*' or SCI or 'central cord syndrome*' or 'upper motor neuron disease*' or parapleg* or quadriplegi* or tetraplegi*) AND TS = ('end effector' or 'end-effector' or 'robot* assisted rehab*' or 'robot* assisted train*' or 'upper extremit* robot*' or 'upper limb* robot*'or 'upper body robot*' or 'lower extremit* robot*' or 'lower limb* robot*'or 'lower body robot*')) AND (TS = (gait or walking or ambulation or locomotion or 'gait apraxia' or 'gait disorder' or 'locomotion disorder*' or 'upper extremit* impairment*' or 'upper limb* impairment*' or 'upper body impairment*' or 'lower extremit* impairment*' or 'lower limb* impairment*' or 'lower body impairment*' or 'upper extremit* function' or 'upper limb* function' or 'upper body function' or 'lower extremit* function' or 'lower limb* function' or 'lower body function') OR TS = ('gait rehabilitation' or 'walking rehabilitation' or 'locomotor rehabilitation' or 'locomotor training' or 'gait training' or 'step training' or 'hand rehabilitation' or 'arm rehabilitation' or 'upper extremit* rehab*' or 'upper limb* rehab*' or 'upper body rehab*' or 'lower extremit* rehab*' or 'lower limb* rehab*' or 'lower body rehab*')) NOT TS = (Exoskeleton* or exosuite* or amput* or 'limb loss' or 'arm loss') – limited to English and articles

S.1.2. Ovid MEDLINE

(((('Spinal Cord' adj3 (injur* or contusion* or trauma* or transection* or laceration* or compression* or damag*)) or SCI or 'central cord syndrome*' or 'upper motor neuron disease*' or parapleg* or quadriplegi* or tetraplegi*).tw,kw.) AND (('end effector' or 'end-effector' or 'robot* assisted rehab*' or 'robot* assisted train*' or ((upper adj3 (extremit* or limb* or body)) and robot*) or ((lower adj3 (extremit* or limb* or body)) and robot*)).tw,kw.) AND ((gait or walking or ambulation or locomotion or 'gait apraxia' or 'gait disorder' or 'locomotion disorder*' or ((upper adj3 (extremit* or limb*)) and function*) or ((upper adj3 (extremit* or limb*)) and impairment*) or ((lower adj3 (extremit* or limb*)) and function) or ((lower adj3 (extremit* or limb*)) and impairment*)).tw,kw.) OR (('gait rehabilitation' or 'walking rehabilitation' or 'locomotor rehabilitation' or 'locomotor training' or 'gait training' or 'step training' or ((upper adj3 (extremit* or

limb* or body)) and rehab*) or ((lower adj3 (extremit* or limb* or body)) and rehab*) or 'hand rehabilitation' or 'arm rehabilitation').tw,kw.)) AND NOT ((Exoskeleton* or exosuite* or amput* or 'limb loss' or 'arm loss').tw,kw.)

S.1.3. Ovid EMBASE

(((('Spinal Cord' adj3 (injur* or contusion* or trauma* or transection* or laceration* or compression* or damag*)) or SCI or 'central cord syndrome*' or 'upper motor neuron disease*' or parapleg* or quadriplegi* or tetraplegi*).tw,kw.) AND (('end effector' or 'end-effector' or 'robot* assisted rehab*' or 'robot* assisted train*' or ((upper adj3 (extremit* or limb* or body)) and robot*) or ((lower adj3 (extremit* or limb* or body)) and robot*)).tw,kw.) AND ((gait or walking or ambulation or locomotion or 'gait apraxia' or 'gait disorder' or 'locomotion disorder*' or ((upper adj3 (extremit* or limb*)) and function*) or ((upper adj3 (extremit* or limb*)) and impairment*) or ((lower adj3 (extremit* or limb*)) and function) or ((lower adj3 (extremit* or limb*)) and impairment*)).tw,kw.) OR (('gait rehabilitation' or 'walking rehabilitation' or 'locomotor rehabilitation' or 'locomotor training' or 'gait training' or 'step training' or ((upper adj3 (extremit* or limb* or body)) and rehab*) or ((lower adj3 (extremit* or limb* or body)) and rehab*) or 'hand rehabilitation' or 'arm rehabilitation').tw,kw.)) AND NOT ((Exoskeleton* or exosuite* or amput* or 'limb loss' or 'arm loss').tw,kw.)

S.1.4. EBSCO – CINHAL

(((('Spinal Cord' N3 (injur* or contusion* or trauma* or transection* or laceration* or compression* or damag*)) or SCI or 'central cord syndrome*' or 'upper motor neuron disease*' or parapleg* or quadriplegi* or tetraplegi*)) AND (('end effector' or 'end-effector' or 'robot* assisted rehab*' or 'robot* assisted train*' or ((upper N3 (extremit* or limb* or body)) and robot*) or ((lower N3 (extremit* or limb* or body)) and robot*))) AND ((gait or walking or ambulation or locomotion or 'gait apraxia' or 'gait disorder' or 'locomotion disorder*' or ((upper N3 (extremit* or limb*)) and function*) or ((upper N3 (extremit* or limb*)) and impairment*) or ((lower N3 (extremit* or limb*)) and function) or ((lower N3 (extremit* or limb*)) and impairment*))) OR (('gait rehabilitation' or 'walking rehabilitation' or 'locomotor rehabilitation' or 'locomotor training' or 'gait training' or 'step training' or ((upper N3 (extremit* or limb* or body)) and rehab*) or ((lower N3 (extremit* or limb* or body)) and rehab*) or 'hand rehabilitation' or 'arm rehabilitation'))) AND NOT ((Exoskeleton* or exosuite* or amput* or 'limb loss' or 'arm loss')))

S.2. Supplementary Data Tables

Table S1: Details of Study Intervention Protocols

Author	Duration	Setting	Frequency	Protocol	End-Effector Device	Fixed/ Personalised/ Adaptive Program
Freivogel, Schmalohr and Mehrholz [28]	6 weeks	Hospital	30 minutes, 3-5 times a week	Patients were randomly allocated to intervention sequence AB or BA. Intervention A involved 20 RAGT treatments. Intervention B involved 20 treatments on the treadmill or practiced walking on the floor.	The LokoHelp (LokoHelp Group, Germany)	Adaptive/ Personalised? The velocity of the treadmill was set to a maximum speed tolerated by the patients (up to 2.5 km/h) and step length was fixed at 400 mm
Benito-Penalva, Edwards [29]	8 weeks or 20 WISCI-II score	Hospital	1 time per day, 5 times per week	Patients received locomotor training exclusively on 1 of the systems, in place of therapist-assisted gait training.	Gait Trainer GT I (Reha-Stim, Germany)	Adaptive
Hesse, Werner and Bardeleben [30]	5 weeks	Neurological Rehab Clinic	1 time per day	20-25 mins of locomotor training associated to FES, in addition to the regular therapy Optional breaks after 5mins	GT I (Reha-Stim, Germany)	Adaptive
Shin, Jeon [31]	4 weeks	Hospital	5 times per week	Patients received 30 mins of RAGT and 1 h of CT, which involved sitting and standing balance training, sit-to-stand training, overground gait training (if possible), and strengthening exercises	Morning Walk (Curexo, Seoul, South Korea)	Adaptive (Cadence was increased by five steps/minute if a patient could perform RAGT for 10 minutes without resting. The step length was modified according to cadence. BWS set to average of previous session.)
Shin, Jeon [32]	4 weeks	Hospital	5 times per week	Patients were randomly allocated to RAGT or CT group RAGT intervention included: 30 mins of RAGT and 1h of CT, with the latter involving 30 mins of ergometer training and 30 mins of sitting and standing balance training, sit-to-stand training,	Morning Walk (Curexo, Seoul, South Korea)	Adaptive (Cadence was increased by five steps/minute if a patient could perform RAGT for 10 minutes without resting. The step length was

				overground gait training (if possible), and strengthening exercises<br>CT intervention included:<br>1.5 h of CT, involving 30 mins of ergometer training and 1h of sitting and standing balance training, sit-to-stand training, overground gait training (if possible), and strengthening exercises		modified according to cadence. BWS set to average of previous session)
Calabrò, Filoni [33]	2 months	Hospital	1 time a day,<br>6 times per week		G-EO System<br>(Reha Technology, Switzerland	Adaptive
Hornby, Kinnaird [35]	Single session	Rehab Centre	6 minutes	1 session with three different devices (treadmill, exoskeleton and end effector).<br>All participants familiarized to stepping in each device for 2 to 4 minutes prior to 6 minutes testing.	Pedago<br>(Lokohelp, Zlin,<br>Czech Republic)	Fixed
Bonnevie, Moily [37]	Single session	Rehab Centre	Between 12 to 29 minutes (median = 18) depending on participants	Patients were given 1 to 2 mins to get accustomed to the movement and ensure they were set up comfortably, to then start the session with the E-RAGT.	LEXO<br>(Tyromotion, GmbH, Austria)	Unknown
Choi, Kim [38]	24 sessions	Hospital	30 minutes,<br>5 times a week	Protocol consisted of 3 phases:<br>screening, initial assessment, main treatment.<br>The screening consisted of 5 mins of walking along a “flat surface” followed by 5 minutes of gait in the “stair-up” mode, and another 5 minutes of gait in the “stair-down” mode.<br>Following the screening patients were then classified into *hard* (>70% BW supported and GRF remained unchanged at <20% BW), *difficult* (>60% BW and needed support from therapist), and *possible prediction groups* (did not fall neither in the hard nor difficult).<br>Main treatment began for a total of 24 sessions.	Morning Walk®<br>(Curexo, Seoul,<br>South Korea)	Unknown
Maggio, Bonanno [39]	2 to 6 months, depending on severity	Hospital	6 days per week	Every week, patients received a comprehensive schedule of five to seven training sessions, including:<br>- 5 to 7 physiotherapy sessions<br>- 6 to 10 robotic treatments<br>- 3 psychological sessions<br>- 2 to 5 speech therapy sessions	G-EO System<br>(Reha Technology, Switzerland)	Unknown

				- 3 to 5 occupational therapy sessions<br>- 1 neuro-sexual consultation		
Upper Limb:						
Mackenzie, Tan and Benad [34]	3 weeks	Hospital	6 sessions of 1 h each in addition to 1h OT	Functional tasks were performed involving:<br>(a) practising brushing teeth<br>(b) upper body dressing which involved putting a t-shirt over the head<br>(c) practising hanging clothes out on a make-shift line above head level	DIEGO (Tyromotion, Austria)	Adaptive
Kilkki, Poutanen [36]	6 weeks	Rehab Centre	3 sessions a week, 1hour each	Minimum 30mins of device use for each session. Device and task use selected individually for each participant. Other 30mins included other required rehabilitation activities.	*AMADEO, and DIEGO* *(*(Tyromotion Inc., Graz, Austria)	Unknown
Vanmulken, Spooren [40]	6 weeks	Rehab Centre	3 days a week for 1 hour	2 sessions of 30 minutes each, interspaced by a 30-min break	Haptic Master (Netherlands)	Unknown
Jung, Lee [41]	5 weeks	Hospital	40 minutes (20 + 20) in addition to 30 minutes OT,<br>3 days a week,<br>1 session per day	Participants were randomly assigned to a RT or OT group.<br>The RT group received, in addition to conventional OT, a combined upper limb RT using an end-effector (20 minutes) and an exoskeleton (20 minutes).	*ArmeoPower (Exoskeleton, Hocoma Inc., Switzerland)*<br>*Hand:* AMADEO (Tyromotion Inc., Graz, Austria),	Unknown
Mackenzie, Tan and Benad [42]	22 days (Monday to Friday)	Hospital/ Community	5 sessions per week	Every week protocol involved:<br>2 sessions of 1 hour each involving traditional upper limb rehabilitation and 3 sessions of 1 hour each with the end-effector	DIEGO (Tyromotion, Austria)	Adaptive

FES = functional electrical stimulation; BWS = bodyweight support; GRF = ground reaction force

Table S2: Lower-Limb Functional Outcome Measures and Results

Author		10MWT (m/s)	6mWT	LEMS	WISCI	SCIM	FAC	BBS	EMG	MAS	ISNCSCI
Freivogel, Schmalohr and Mehrholz [28]<br>Δ Mean (Δ SD)		X					X	X		X	
Benito-Penalva, Edwards [29]<br>Mean ± SE	Initial	0.094±0.207		21.82±1.37	4.11±0.61						
	Final	0.277±0.041		31.12±1.45	9.23±0.86						
Hesse, Werner and Bardeleben [30]		1-0.32m/s-0.81m/s<br>2-0.12m/s(aided)-0.26m/s(AFO)<br>3-0-0.15m/s(aided)<br>4-0.16m/s(aided)-0.65m/s(AFO's)	1 – 34m-296m<br>2-14m(aided)-99m(AFO)<br>3-0-17m(aided)<br>4-13m(aided)-112m(AFO's)						RF, G, TA, VM		
Shin, Jeon [31]<br>Median (IQR)	ρ	ρ=0.002	ρ=0.002	ρ=0.003	ρ=0.001			ρ=0.001			ρ=0.027
	Initial	0(0-0)	0(0-0)	34(30-37)	3(0-4)			14 (8–26)			10 (6–12)
	Final	0.16(0.12-0.5)	52(38-129)	39(38-40)	13(9-19)			38 (24–49)			12 (10–12)
Shin, Jeon [32]<br>Mean (IQR)	ρ	ρ=0.001	ρ=0.001	ρ=0.001	ρ<0.0001	ρ=0.001		ρ<0.0001			ρ=0.007
	Initial	0(0-0.1)	0(0-24)	35.0(29.8-38.0)	3.5(0.8-6.5)	5.0(3.0-11.2)		12.5(8.0-21.5)			9.5(6.0-12.0)
	Final	0.2(0.1-0.5)	53.8(35.5-140.5)	38.5(37.8-40.0)	13.0(12.0-19.2)	13.5(7.2-18.2)		38.5(22.5-49.5)			12.0(9.8-12.0)
Calabrò, Filoni [33]		ρ=0.004			ρ=0.004	ρ<0.001			VL, BF, TA, GM	X	
Hornby, Kinnaird [35]				X	X				LEFT LIMB		
		Measured but as confounding variables against kinematics and EMG rather than output. No outputs of improvement as repeated measures study.									
Choi, Kim [38]<br>Median (IQR)	ρ						ρ<0.001				
	Pre						1.7 (0.5-3.0)				
	Post						3.0(2.0-4.0)				

X - Outcome measure included but no usable reported result, EMG – Electromyography measure of muscle activity

Table S3: Upper-Limb Functional Outcome Measures and Results

Article	Result Type		Functional Outcome Measures: UEMS	SCIM		Other			
**Mackenzie, Tan and Benad [34]**	Test Spec		-	Self-care		COPM: Cleaning Teeth		Visual Analogue Scales	
						Satisfaction	Performance	Fatigue	Pain
	Pre			0		1	1	4	2
	Post			4		4	3	2	2
**Kilkki, Poutanen [36]**	Median Difference (IQR) pre-post	Group		Total	Self-care	Action Research Arm Test			
		AB	Δ1 (0 – 2)	Δ0 (0 – 0)	Δ0 (0 – 1)	Δ0 (-1 – 1)			
		BA	Δ0 (-2 – 0)	Δ0 (-6 – 1)	Δ1 (-1 – 0)	Δ0 (0 – 3)			
**Vanmulken, Spooren [40]**			-	“No discernible differences”		Van Lieshout Test<br>P2 and P5 progressed by 1 point on the scale (0-5).<br>P4 had a baseline at maximum.			
**Jung, Lee [41]**	Test Spec		Total (C5, C6, C7, C8, T1)	Self-care		GRASSP Prehension			
	ρ-Value		ρ=0.018	ρ=0.008		ρ=0.107			
	Pre (Mean ± SD)		14.59±6.49	5.88±5.34		13.47±11.27			
	Post Mean ± SD		16.18±7.25	8.23±6.74		14.58± 11.39			
**Mackenzie, Tan and Benad [42]**	Test Spec			Total	Self-care	COPM		Visual Analogue Scales	
						Satisfaction	Performance	Satisfaction	Performance
	ρ-Value		-	ρ=0.01	-	ρ=0.028	ρ=0.028	ρ=0.028	ρ=0.028
	Pre			14	0	0.80	0.84	0.80	0.84
	Post			17.8	1.14	3.32	2.96	3.32	2.96

Table S4: Upper-Limb Strength Outcome Measures and Results

Article	Result Type	Strength Outcome Measures				
Mackenzie, Tan and Benad [34]	Test Spec	Elbow				Shoulder
		Flexion		Extension		All
		R	L	R	L	
	Pre	5	5	2	5	5
	Post	5	5	3	5	5

Article	Result Type	Grip Strength		2-point Pinch		3-point Pinch		Lateral Pinch	
Kilkki, Poutanen [36]	Median Difference (IQR) pre-post	R	L	R	L	R	L	R	L
		Δ0 (-2 – 2)	Δ1 (-4 – 2)	Δ0 (-1 – 0)	Δ0 (0 – 2)	Δ0 (-2 – 2)	Δ1 (-4 – 2)	Δ0 (-1 – 0)	Δ0 (0 – 2)
		Δ0 (-2 – 3)	Δ2 (-2 – 2)	Δ0 (0 – 0)	Δ0 (-1 – 0)	Δ0 (-2 – 3)	Δ2 (-2 – 2)	Δ0 (0 – 0)	Δ0 (-1 – 0)

Article	Result Type	Strength Outcome Measures	
Vanmulken, Spooren [40]		The International Classification for Surgery of the Hand in Tetraplegia and MicroFet_2 (Hoggan Health Industries, Salt Lake City, UT, USA) strength measurement.<br><br>No changes for any participants.	P2 improved on all arm strengths (8-22%)<br>P5 elbow flexion +23%, shoulder abduction +13%, wrist extension +17%<br>P4 no improvements.

Article	Result Type				
Jung, Lee [41]	Test Spec	Full Grasp	Tip Pinch	Lateral Pinch	Three-Jaw Chuck
	ρ-Value	ρ=0.213	ρ=0.798	ρ=0.878	ρ=0.624
	Pre Mean ± SD	4.26±4.54	0.91±0.97	2.28±2.32	1.52±1.52

Article	Result Type	Elbow				Shoulder									
Mackenzie, Tan and Benad [42]	Test Spec	Flexion		Extension		Flexion		Extension		Internal Rotation		External Rotation		Abduction	
		R	L	R	L	R	L	R	L	R	L	R	L	R	L
	ρ-Value	ρ=0.180	ρ=0.317	ρ=0.059	ρ=0.180	ρ=0.109	ρ=0.060	ρ=0.180	ρ=0.157	ρ=0.655	ρ=0.157	ρ=0.655	ρ=0.157	ρ=1.000	ρ=0.068
	Pre	1.71	2.00	0.86	1.00	1.29	1.57	2.00	1.71	2.14	2.14	2.00	2.00	2.00	1.57
	Post	2.12	2.29	1.86	2.00	2.29	2.43	2.29	2.14	1.57	2.71	1.43	2.29	2.17	0.86

Table S5: Upper-Limb Range of Motion Outcome Measures and Results

Article	Result Type	Range of Motion Outcome Measures													
**Mackenzie, Tan and Benad [34]**	Test Spec	Elbow													
		Flexion		Extension		Pronation									
		R	L	R	L	R	L								
	Pre	140	131	Full	Full	60	131								
	Post	146	150	Full	Full	125	140								
**Kilkki, Poutanen [36]**															
**Vanmulken, Spooren [40]**															
**Jung, Lee [41]**															
**Mackenzie, Tan and Benad [42]**	Test Spec	Elbow				Shoulder									
		Flexion		Extension		Flexion		Extension		Internal Rotation		External Rotation		Abduction	
		R	L	R	L	R	L	R	L	R	L	R	L	R	L
	ρ-Value	ρ=0.109	ρ=0.180	ρ=0.317	ρ=1.000	ρ=0.042	ρ=0.068	ρ=0.180	ρ=0.317	ρ=0.043	ρ=0.109	ρ=0.273	ρ=0.180	ρ=0.109	ρ=0.180
	Pre	71.00	63.00	-3.86	0.00	29.00	26.86	14.29	17.86	37.71	34.14	27.14	23.57	34.71	26.86
	Post	82.29	67.50	-15.33	0.00	39.57	48.29	16.87	15.83	36.00	32.67	33.00	26.00	39.57	46.00

Pre – pre-therapeutic protocol result, Post – post-therapeutic protocol result, R – right, L – left.
